# Confinement to the intrapancreatic bile duct is independently associated with a better prognosis in extrahepatic cholangiocarcinoma

**DOI:** 10.1186/s12876-016-0444-1

**Published:** 2016-02-24

**Authors:** Keun Soo Ahn, Koo Jeong Kang, Yu Na Kang, Yong Hoon Kim, Tae-Seok Kim

**Affiliations:** Department of Surgery, Keimyung University School of Medicine, Dongsan Medical Center, 56 Dalsung-ro, Jung-gu, Daegu City, Republic of Korea; Department of Pathology, Keimyung University School of Medicine, Dongsan Medical Center, 56 Dalsung-ro, Jung-gu, Daegu City, Republic of Korea

**Keywords:** Extrahepatic cholangiocarcinoma, Location, Prognosis

## Abstract

**Background:**

Actual differences of long term outcome of extrahepatic cholangiocarcinoma according to the location of the tumor have not yet been studied. The aim of this study was to evaluate the prognosis and optimal surgical procedure for middle (BD) cancer.

**Methods:**

Among 109 patients with carcinoma of the extrahepatic BD underwent surgical resection, curative resection of extrahepatic BD cancer was performed in 90 patients. They were classified into three groups according to the location of tumors: DISTAL (*n* = 32), tumor was confined to the intrapancreatic bile duct; MID (*n* = 20), tumor was located between below the confluence of the hepatic duct bifurcation and suprapancreatic portion of the BD; and DIFFUSE (*n* = 38), tumor was located diffusely.

**Results:**

Tumor involving the middle BD (MID or DIFFUSE) had a higher rate of perineural invasion as compared to the DISTAL group. The overall and disease-free survival rate for the MID or DIFFUSE group was significantly worse than that of DISTAL. In the MID/DIFFUSE group, there was no significant difference of survival according to the type of the operation (pancreaticoduodenectomy or segmental BD resection). The multivariate analysis showed that tumor involving middle BD (MID or DIFFUSE group) and node metastasis were independently poor prognostic factors for the disease free and overall survival.

**Conclusion:**

Extrahepatic cholangiocarcinoma involving the extrapancreatic BD has a worse prognosis than those confined to the intrapancreatic BD. In patients with tumors confined to the middle BD, BD resection can be considered as an alternative surgical procedure to pancreaticoduodenectomy, if an R0 resection can be accomplished.

## Background

Extrahepatic cholangiocarcionoma is classified into perihilar cholangiocarcinoma and distal bile duct (BD) cancer according to the anatomical location of the tumor as defined in the 7^th^ edition of American Joint Committee on Cancer’s (AJCC) classification [1, 2]. For convenience, distal BD cancer has been further subdivided into middle BD carcinoma, which is defined as the infrahilar/suprapancreatic area (or from the confluence of cystic duct to the superior border of pancreas) and distal BD cancer, which is referred to as intrapancreatic BD [3–6]. However, an exact location of the middle BD is difficult to define because of different locations of the cystic duct confluence. Additionally, tumors are rarely confined to one segment (proximal, middle, or distal BD) microscopically, and bile duct cancer tends to spread along the bile duct epithelium longitudinally, rather be than confined to one area [2, 7, 8]. While there are different operative approaches between hilar cholangiocarcionma and extrahepatic bile duct cancer, pancreaticodudenectomy (PD) has been regarded as a standard operative procedure for mid and distal BD cancer, although segmental BD resection can be applied in limited patients. For these reasons, in recent literature, middle and distal BD cancer were classified together as ‘distal bile duct’ cancer and have a same AJCC 7^th^ classification.

However, middle BD cancer has a different behavior when compared to distal BD cancer. In cancer confined to the intrapancreatic portion, periampullary structures, such as the pancreas or duodenum, may limit the tumor spread into adjacent tissues. In contrast, cancer located in the middle BD may have a higher possibility of earlier microscopic tumor invasion of the periductal structure in the hepatodudenal ligament. This may affect the encasement of the adjacent hepatic artery or portal vein earlier, and result in a higher chance of radial margin involvement. However, although the possibility of poor outcomes in middle CBD cancer have been suggested, actual differences of long term outcome according to the location have not yet been studied [5, 9–13] and the study which showed prognostic impact of the primary tumor site is rare [14]. Furthermore, the appropriate operative procedure for tumors confined to the middle BD has not been fully defined. Although usually, PD is needed for curative resection in mid BD cancer, there has been debate about whether segmental resection of the bile duct can be an alternative treatment or not when negative margins can be obtained [15].

Our hypothesis was that BD cancer involving the middle BD, may show a worse prognosis than cancer confined to the intrapancreatic BD. For the treatment of cancer confined to the mid BD, the type of surgical procedure (PD or segmental resection of BD) may not affect the survival because tumor location in the mid BD may be significantly poor prognostic factor regardless of operative type. If this is true, segmental resection of the BD in tumors confined to the mid BD would be justified. Therefore, the aim of this study was to analyze the clinicopathological factors that influence the survival of extrahepatic BD cancer according to the location of the tumor, and to determine the oncologic safety of segmental BD resection as an optimal surgical procedure for a cancer confined to the middle BD.

## Method

### Population and study design

**Fig. 1:**
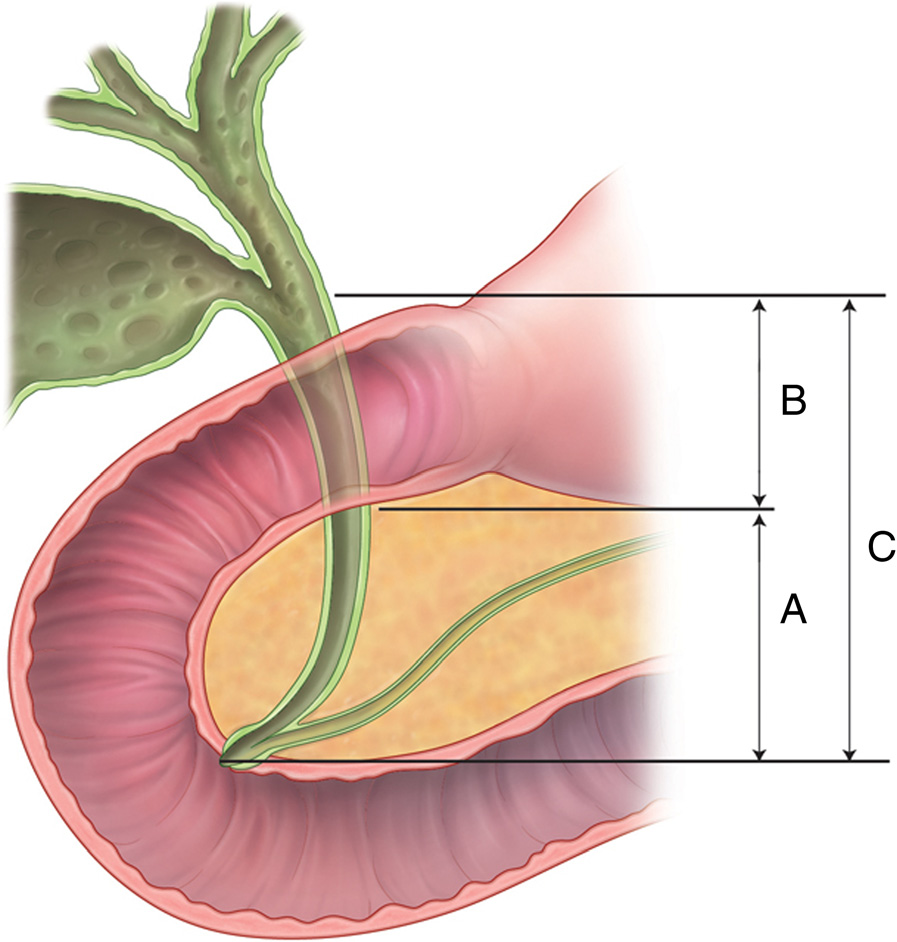
Classification into the three groups according to anatomical location of the tumor based on histopathological findings; the DISTAL group (**a**), tumors confined to the intrapancreatic BD; the MID group (**b**), tumors confined to the middle bile duct; and the DIFFUSE group (**c**), tumors located in the diffusely middle and intrapancreatic BD

From March 2000 to August 2012, 110 patients with adenocarcinoma of the distal BD underwent surgical resection at the Keimyung University Dongsan Medical Center. Eighteen of the patients were unable to undergo curative R0 resection; they either underwent an R1 or R2 resection or had distant metastases. These patients and additional two patients with in-hospital mortality were excluded. The remaining 90 patients who had extrahepatic cholangiocarcinoma originating from the middle or distal bile duct histologically confirmed with negative longitudinal or radial margin (R0) were enrolled in this study. The 90 patients were classified into three groups according to the anatomical location of the tumor based on histopathological findings: 1) the distal BD cancer group (DISTAL group), tumor was confined to the intrapancreatic BD 2) the middle BD cancer group (MID group), tumor was located between the confluence of the hepatic duct bifurcation and suprapancreatic portion of BD; and 3) the diffuse BD cancer group (DIFFUSE group), tumor was located diffusely within the middle and intrapancreatic BD (Fig. 1). When the main tumor was located in the intrapancreatic portion of the BD, if tumor cells were present at the mid bile duct in microscopic examination, the lesion was classified as DIFFUSE group. Patients’ demographics, perioperative outcomes, tumor histopathology, and follow-up data were analyzed retrospectively based on a prospectively recorded database. This retrospective study protocol was reviewed and approved by the institutional review board of the Keimyung University Dongsan Medical Center (11–308). The consents from patients for this retrospective study were waived by the institutional review board.

### Preoperative management, surgical procedure and histopathological evaluation

There were no absolute criteria for performing preoperative biliary drainage. In general, if patients showed jaundice with bilirubin levels higher than 10 mg/dl or if a biopsy was necessary, biliary drainage endoscopically or percutaneously was performed.

The surgical procedure was determined by the extent of the lesion. All patients in the DISTAL and DIFFUSE groups underwent a standard PD or pylorus-preserving pancreaticoduodenectomy (PPPD). In the MID group, a segmental BD resection with hepaticojejunostomy was performed when a negative margin could be obtained, and PD was performed when obtaining of negative margin was not assured preoperatively or intraoperatively. A routine resection of the lymph nodes around the porta hepatis (No.12), retropancreatic (No.13), common hepatic artery and celiac trunk (No 8,9) was done.

During the study period, one pathologist reviewed the pathology to verify whether each sample was feasible for this study on the basis of the histopathologic diagnoses recorded and accumulated. Tumor stages were classified according to the 7^th^ edition of AJCC. Postoperative surgical complications were graded as described by the proposed Dindo-Clavien classification system [16].

### Follow up and diagnosis of recurrence

The routine follow-up program consisted of a physical examination, computed tomography, and tumor markers (carcinoembryonic antigen (CEA), carbohydrate antigen (CA) 19–9) every 3 mohths for the first year, then every 6 months for the next 4 years, and thereafter, annually.

### Statistical analysis

A statistical analysis were performed using SPSS, version 13.0 for Windows (SPSS,Inc., Chicago IL,USA). Comparison between the groups was performed using an independent *t* test and ANOVA for continuous variables and Chi-square test for categorical variables. Overall and disease-free survivals were calculated using the Kaplan-Meier method. A univariate analysis of the survival curves was performed using the log-rank test. A multivariate regression analysis was performed using a Cox proportional hazards model to identify the independent prognostic factors for survival. A *P* value of <0.05 was considered statistically significant.

## Results

### Demographic, perioperative and histopahological results

The patient population consisted of 38 women and 52 men, with a mean age of 65 years (range, 33–83 years). These patients were grouped as follows: 32 patients of the DISTAL group (35.6 %), 20 of the MID group (22.2 %) and 38 of the DIFFUSE (42.2 %). In the MID group, five patients (25.0 %) underwent PPPD, and the remaining 15 patients underwent BD resection. The demographic factors, perioperative results, and histopathological results showed no differences between three groups. However, a higher rate of T3 was observed in the DISTAL and DIFFUSE groups than the MID group. In the DISTAL group, rate of perineural invasion was lower than that of the MID and DIFFUSE groups (Table 1).

**Table 1:** Perioperative and histopathological characteristics between three groups

	DISTAL (*n* = 32)	MID (*n* = 20)	DIFFUSE (*n* = 38)	*P* value
Age (years)	65.1 ± 9.7	64.3 ± 9.6	67.1 ± 6.7	0.545
Gender (male/female)	18/14	13/7	21/17	0.757
Preoperative peak TB (mg/dl)	7.0 ± 6.2	8.9 ± 9.4	6.5 ± 4.8	0.629
CA 19–9 ≥ 37	21 (70.0 %)	15 (62.0 %)	25 (68.6 %)	0.932
Preoperative biliary drainage (n)	25 (83.3 %)	19 (82.6 %)	32 (84.2 %)	0.713
Type of resection				<0.001
Pancreaticoduodenectomy	32 (100 %)	5 (25.0 %)	38 (100 %)	
Bile duct resection	0 (0 %)	15 (75.0 %)	0 (0 %)	
Operative time (min)	419.0 ± 79.8	357.5 ± 106.4	421.3 ± 69.0	0.033
Transfusion (n)	6 (18.8 %)	2 (10.0 %)	5 (13.1 %)	0.524
Postoperative complication (n)	14 (43.8 %)	5 (25.0 %)	18 (47.4 %)	0.431
Grade (I,II/III,IV)	5/9	1/4	6/12	
Postoperative hospital stay (days)	29.4 ± 13.3	23.2 ± 14.2	28.6 ± 13.1	0.157
Gross tumor appearance				0.915
Infiltrating type	26 (81.3 %)	16 (80.0 %)	31 (81.6 %)	
Papillary/nodular type	6 (28.7 %)	4 (20.0 %)	7 (18.4 %)	
Differentiation				0.295
WD	7 (21.9 %)	5 (25.0 %)	6 (15.8 %)	
MD	18 (56.3 %)	6 (30.0 %)	21 (55.3 %)	
PD	7 (21.9 %)	9 (45.0 %)	11 (28.9 %)	
Depth of invasion				0.002^a^
T1	5 (15.6 %)	6 (30.0 %)	9 (23.7 %)	
T2	3 (9.4 %)	9 (45.0 %)	4 (10.5 %)	
T3	24 (75.0 %)	5 (25.0 %)	25 (65.8 %)	
Lymph node metastases	10 (31.3 %)	11 (55.0 %)	13 (34.2 %)	0.191
Stage (AJCC 7^th^)				0.631
Ia/Ib	6 (18.7 %)	9 (45.0 %)	9 (23.7 %)	
IIa/IIb	26 (81.6 %)	11 (55.0 %)	29 (76.3 %)	
Lymphovascular invasion	22 (68.8 %)	11 (55.0 %)	29 (76.3 %)	0.249
Perineural invasion	15 (46.9 %)	16 (70.0 %)	33 (86.8 %)	0.008^b^
Recurrence (n)	13 (40.6 %)	13 (65.0 %)	30 (78.9 %)	0.010^c^
Pattern of initial recurrence				0.449
Local recurrence	4 (30.8 %)	4 (30.7 %)	11 (35.5 %)	
Distant metastases	6 (46.2 %)	4 (30.7 %)	6 (17.2 %)	
Both (local + distant metastases)	3 (23.1 %)	5 (38.4 %)	14 (48.3 %)	

*Abbreviation*: *TB* total bilirubin, *CA* cancer antigen, *WD* well differentiation, *MD* moderate differentiation, *PD* poor differentiation

^a^In comparison between T2 and T3, the rate of T2 was higher and rate of T3 was lower in the MID group than those of the DISTAL and DIFFUSE groups

^b^The rate of perineural invasion was lower in the DISTAL group than that of the MID and DIFFUSE groups

^c^The rate of recurrence was lower in the DISTAL group than that of the MID and DIFFUSe groups

### Survival analysis according to the location, distal vs. mid vs. diffuse, of the tumor

**Fig. 2:**
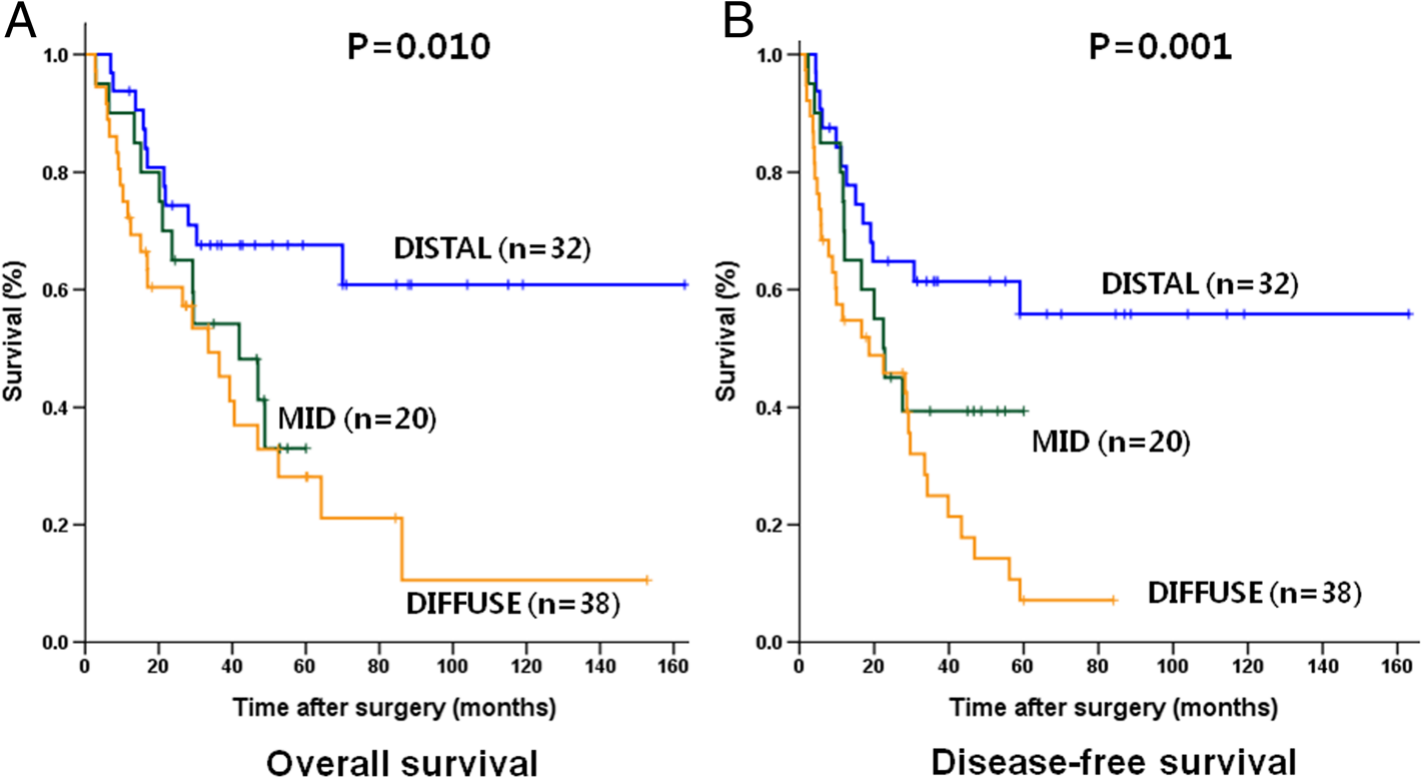
Overall (**a**) and disease-free survival (**b**) in the 3 groups

During the mean follow-up period of 38.8 ± 30.0 (range: 4.5–163.0) months, the 3- and 5- year overall survival rates for the 90 patients were 55.6 and 43.6 %, respectively. The 3- and 5-year disease-free survival rates were 41.6 and 29.9 %, respectively. The overall and disease-free survival rate for the DISTAL group was significant higher than that of the MID and DIFFUSE groups (*P* = 0.010 and 0.001, respectively. Fig. 2). The pattern of recurrence was not different among the 3 groups (Table 1).

**Fig. 3:**
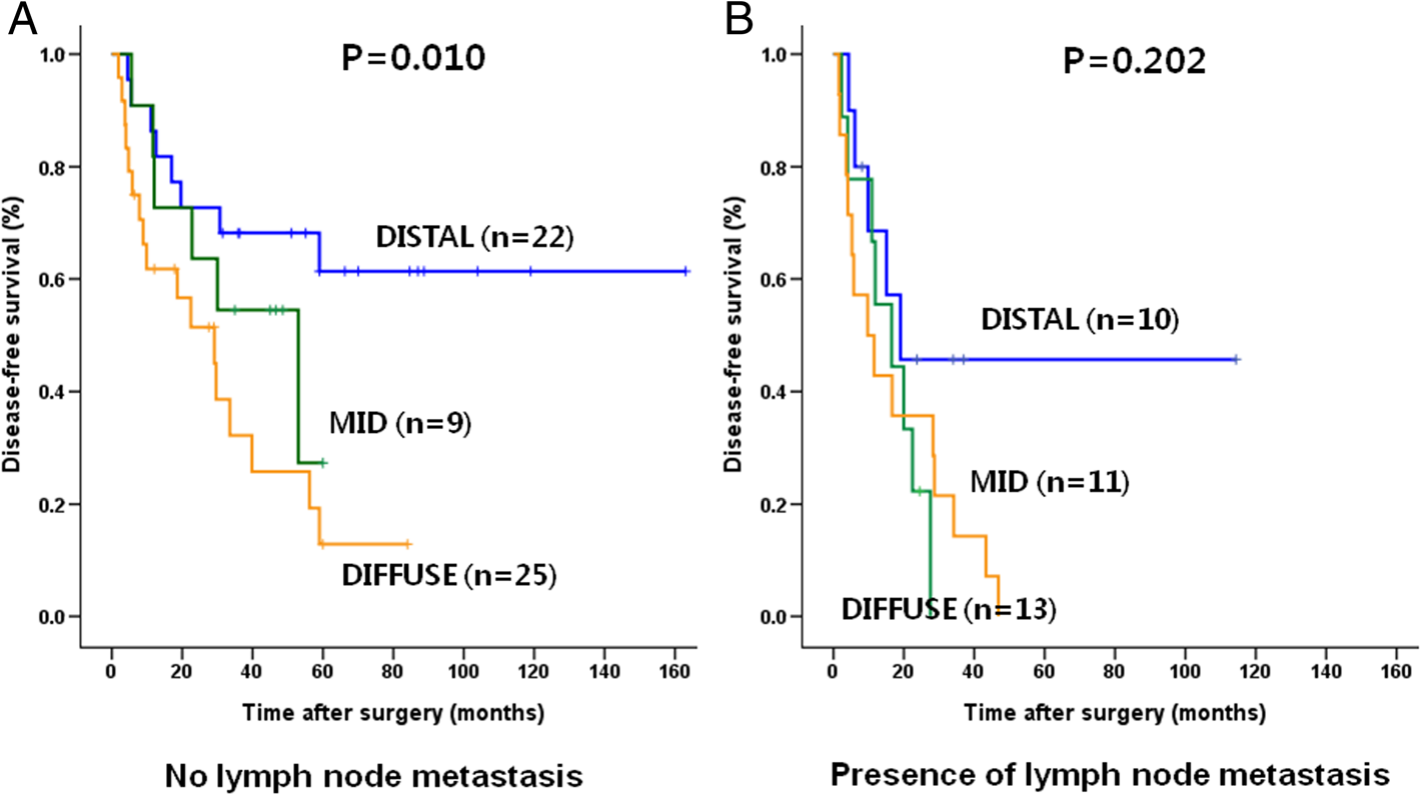
Comparison of the overall survival rates between the DISTAL and the MID/DIFFUSE in IIa (T3N0; **a**) and IIb (AnyTN1; **b**) stage

**Fig. 4:**
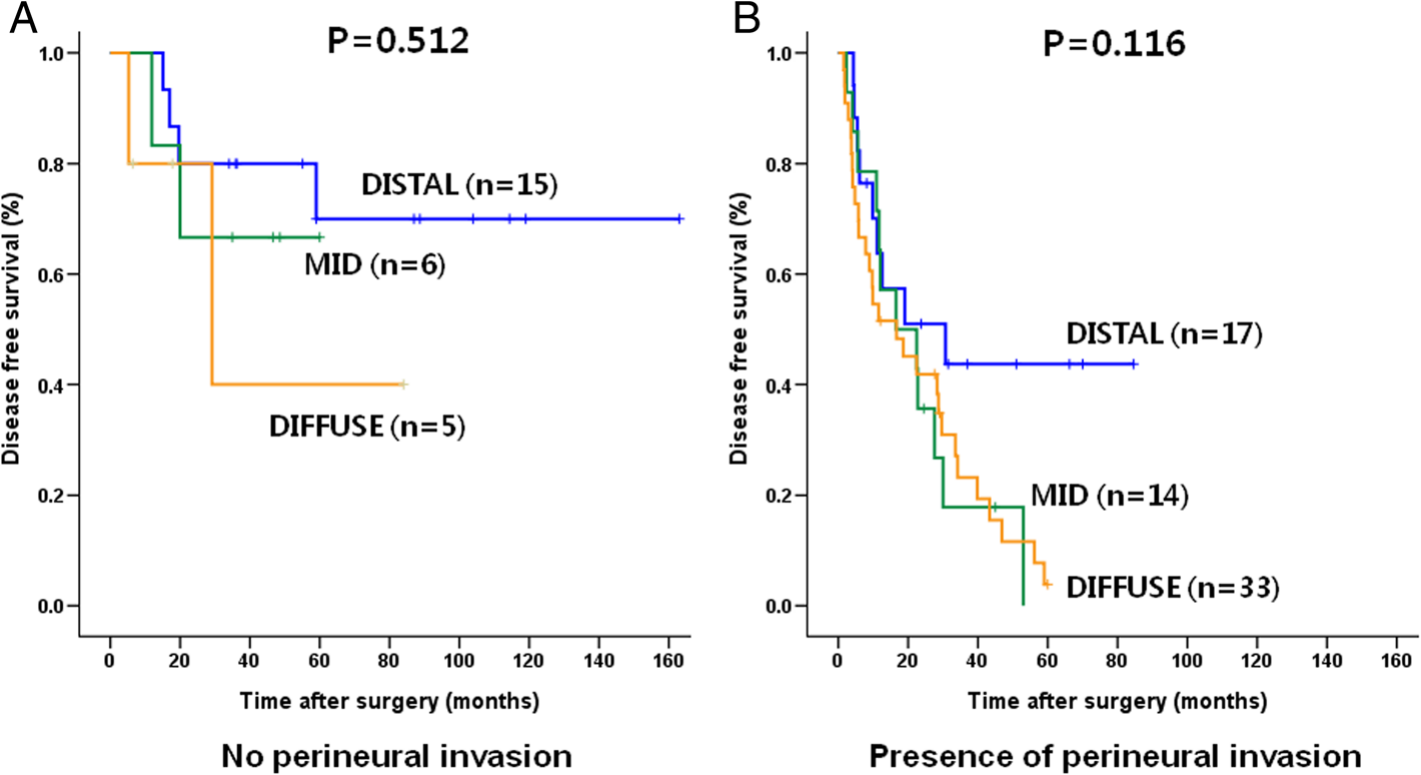
Comparison of the overall survival rates between the DISTAL and the MID/DIFFUSE in presence of perineural invasion (**a**; Absence of perineural invasion, **b**; Presence of perineural invasion)

Further survival analysis, according to the stage and presence of perineural invasion, was done among the three groups. While there was no significant survival difference among the three groups in N1 patients (*P* = 0.202), in N0 patients (including stage IIa (T3N0)), patients of the DISTAL group showed a significantly better survival rate than that of the MID and DIFFUSE group. (*P* = 0.010, Fig. 3) There was no significant survival difference between the DISTAL and MID/DIFFUSE groups depending on the presence of perineural invasion (Fig. 4).

### Survival difference according to the type of resection, segmental resection vs. PD, of the middle bile duc carcinomat

**Fig. 5:**
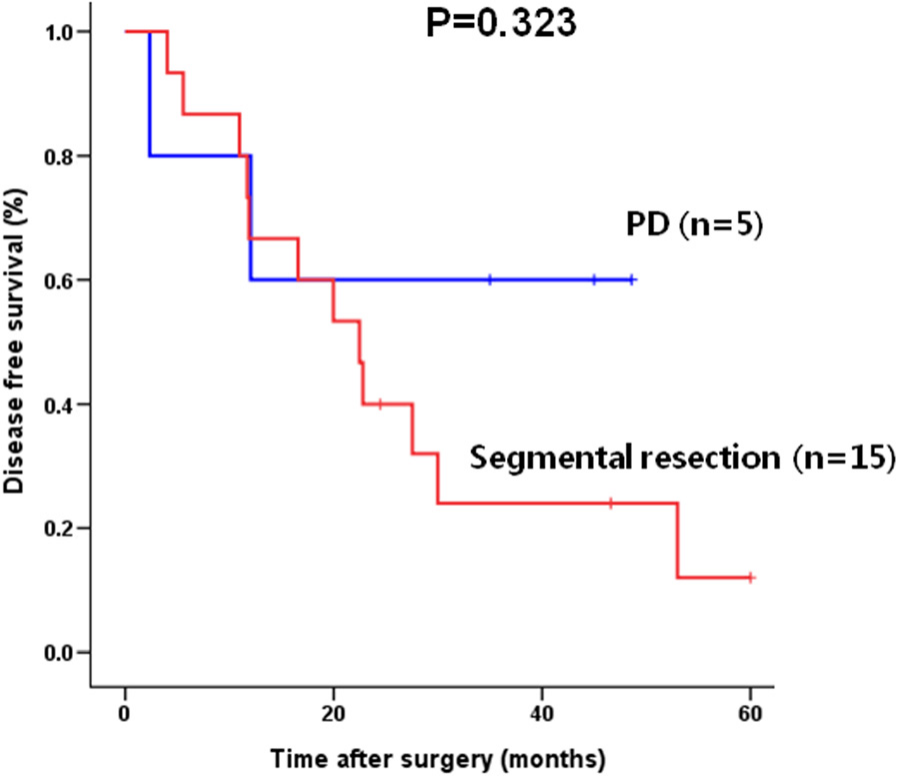
Comparison of the overall survival rates in the MID/DIFFUSE groups according to the type of operation (PD(pancreaticoduodenectomy) vs segmental bile duct resection)

In the MID/DIFFUSE group, there was no difference of disease-free and overall survival according to the type of procedure (PD vs. segmental BD resection, *P* = 0.808). In the MID group, disease-free and overall survival was not different between PD (*n* = 5) and segmental BD resection (*n* = 15) (*P* = 0.323, Fig. 5).

### Prognostic factor of extrahepatic cholangiocarcinoma

An univariate analysis showed that tumor location, gross tumor appearance, differentiation, lymph node metastasis, lymphovascular invasion, and perineural invasion were significant prognostic factors for disease-free and overall survival. The results of the multivariate analysis showed that tumor involving extrapancreatic BD and lymph node metastasis were independently poor prognostic factors for disease-free and overall survival. (Table 2).

**Table 2:** Univariate and multivariate analysis of prognostic factors for overall survival

Variable	5-year overall survival (Univariate analysis)		Multivariate analysis		
	%	*P*	*P*	RR	Exp
Sex		0.901			
Male (*n* = 52)	35.3 %				
Female (*n* = 38)	34.3 %				
Age (years)		0.738			
≤60 (*n* = 35)	34.7 %				
>60 (*n* = 55)	35.2 %				
Location		0.004	0.039		
DISTAL (*n* = 32)	67.6 %			1	(Reference)
MID/DIFFUSE (*n* = 58)	30.2 %			2.299	1.044-5.065
Type of procedure		0.298			
PD/PPPD (*n* = 73)	31.8 %				
Bile duct resection (*n* = 15)	26.7 %				
Transfusion		0.808			
No (*n* = 77)	32.1 %				
Yes (*n* = 13)	26.7 %				
Postoperative complication		0.551			
No (*n* = 53)	40.5 %				
Yes (*n* = 37)	33.7 %				
Preoperative CA 19–9		0.118			
<37 (*n* = 30)	60.4 %				
≥37 (*n* = 60)	36.4 %				
Gross tumor appearance		0.039			
Papillary (*n* = 17)	55.0 %				
Infiltrating (*n* = 73)	34.2 %				
Differentiation		0.028			
WD (*n* = 18)	71.8 %				
MD/PD (*n* = 72)	34.7 %				
Depth of invasion		0.104			
T1 (*n* = 20)	68.8 %				
T2 (*n* = 16)	42.1 %				
T3 (*n* = 54)	34.1 %				
Node metastasis		0.001	0.007		
No (*n* = 56)	51.6 %			1	(Reference)
Yes (*n* = 34)	20.0 %			2.381	1.267–4.475
Lymphovascular invasion		0.050			
No (*n* = 28)	57.2 %				
Yes (*n* = 62)	30.2 %				
Perineural invasion		0.003			
No (*n* = 26)	68.0 %				
Yes (*n* = 64)	33.1 %				
Adjuvant CTx		0.432			
No (*n* = 59)	39.4 %				
Yes (*n* = 31)	37.9 %				

*Abbreviation*: *WD* well differentiation, *MD* moderate differentiation, *PD* poor differentiation

## Discussion

In the present study, patients with extrahepatic cholangiocarcinoma confined to the intrapancreatic portion of BD (DISTAL group) had better survival than those tumors involving the extrapancreatic BD (MID and DIFFUSE group) and it was independent prognostic factor. In the MID/DIFFUSE group, perineural invasion was significantly more frequent. The perineural space is believed to be a separate channel from the lymphatic system, and it can act as a route for tumor metastasis [17]. Clinically, several studies showed that, in patients with biliary tract cancer, including extrahepatic and intrahepatic cholangiocarcionoma and GB cancer, perineural invasion is a significantly poor prognostic factor [18–20]. In tumors involving the mid BD, the tumor may infiltrate the adjacent structures in the hepatoduodenal ligament via neural pathways easily, and the rate of perineural invasion may be more frequent. Invasion of cancer into this area can progress to the surrounding structures, including regional lymph nodes, Glisson’s sheath of the liver, and para-aortic fatty tissue. However, in tumors confined to the intrapancreatic BD, the pancreas or duodenum can act as a barrier of invasion to the surrounding perineural space, and this may be related to the less frequent perineural invasion in the IPBD group. In the present study, although perineural invasion was a significant prognostic factor under the univariate analysis, it was not an independent significant prognostic factor in multivariate analysis. The survival rate between DISTAL and MID/DIFFUSE according to the perineural invasion was not different (Fig. 4). Therefore, perineural invasion may be related to the location of the tumor, and more frequent perineural invasion in the MID/DIFFUSE group may result in a worse prognosis than that of the DISTAL group.

**Fig. 6:**
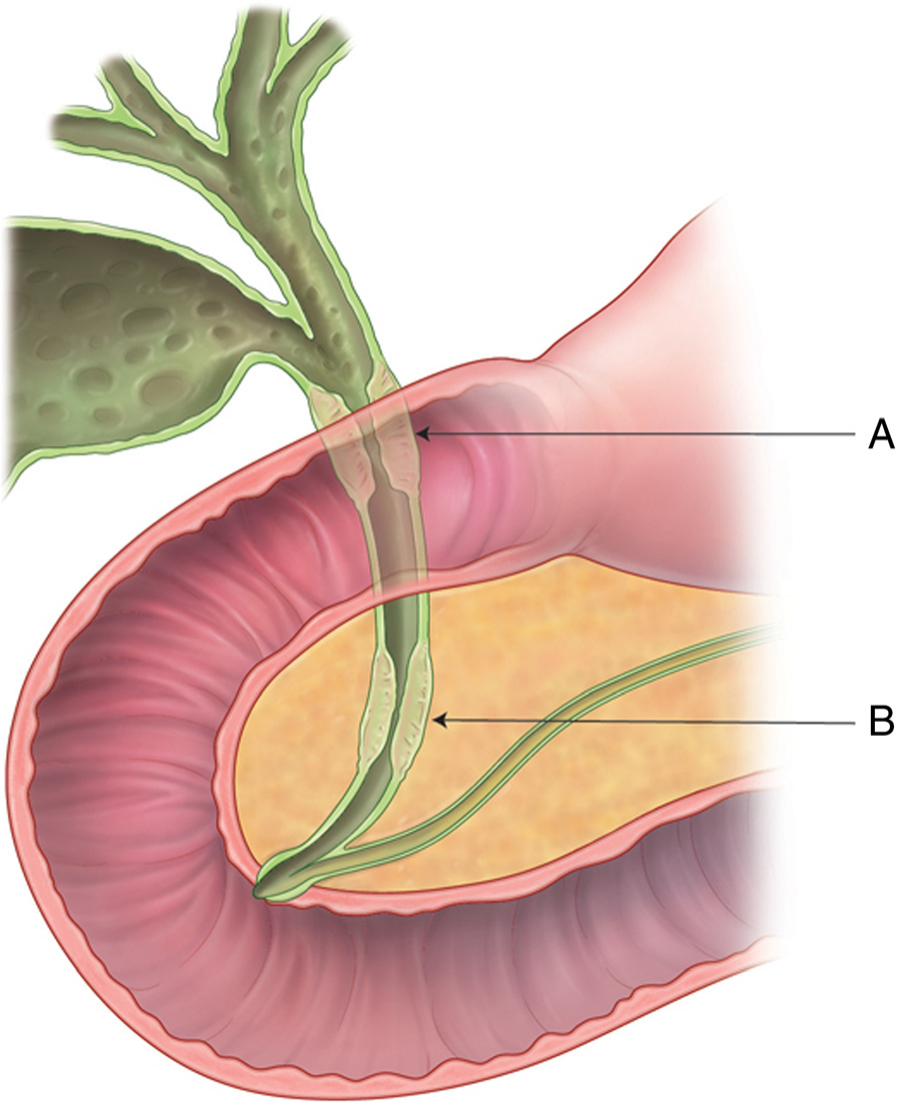
An example of discrepancy of T stage according to the location. Even though two separate tumors have same depth of invasion, tumor involved middle BD cancer (A; MID/DIFFUSE group) would be classified as T2, while tumor confined to intrapancreatic portion of BD (B; DISTAL group) would be classified as T3 because of pancreas invasion

The other factor of different prognosis according to the tumor location is the T stage. Different survival according to the IIa (T3N0) stage between the two groups (Fig. 3) can be explained by the different anatomic structures in the different portions of the extrahepatic BD. Under the current staging system, even if tumors of the distal and middle BD have the same numeric depth of invasion, the T stage is staged differently. Whereas in distal BD cancer, the bile duct is within the head of the pancreas and duodenum; in mid BD cancer, adjacent organs such as the gallbladder, pancreas, and duodenum are far from the BD [21, 22]. A mid BD cancer with the extended pericholedochal soft tissue can be staged as T2 (beyond the wall), while a tumor of the same depth of invasion can be staged as T3 in the distal BD due to invasion of the pancreas. Therefore, a T2 stage of tumor in mid BD cancer may be classified as T3 in distal BD cancer in spite of the same numeric depth of invasion (Fig. 6). A few reports revealed that pancreatic and duodenal invasion does not significantly affect the survival if curative resection is performed [11]. Moreover, smooth muscle fibers in the distal BD have a tendency to form either a continuous or an interrupted layer; whereas, the mid portion of the BD generally has no fibers or only scattered muscle fibers. This thicker smooth muscle bundle of distal BD can also affect the discrepancy of T staging in mid and distal BD cancers [21, 22]. Considering these factors, there is a possibility of T stage underestimation in mid BD cancer compared to distal BD cancer in the same numeric length of the depth of invasion [21]. Therefore, the necessity of alternative T staging has been suggested measuring the actual depth of invasion on a millimeter scale regardless of the adjacent organ invasion to more accurately stage the cancer [21, 23, 24].

In the present study, lymph node positivity, one of the significant prognostic factors [25] in a multivariate analysis, was not related to the location of the tumor. Whereas there was no significant survival difference between the DISTAL and MID/DIFFUSE groups in N1 patients (stage IIb), there was a significance regarding better survival in the DISTAL group in N0 patients (Fig. 3). Therefore, in N0 patients, impact of tumor location and perineural invasion may be magnified in survival.

Our study showed different survival results according to the location of the BD using a different approach. First, we classified extrahepatic BD cancer as DISTAL (confined to intrapancreatic BD) and MID/DIFFUSE (involving extrapancreatic BD). Previous studies have classified middle BD cancer as tumors confined to the mid BD (MID group in our study) which could be treated with BD resection, and distal BD cancer as involvement with the intrapancreatic portion of the BD or diffuse BD cancer (DISTAL and DIFFUSE group in this study), in which PD was necessary [9, 15]. In others, tumors were classified by the predominant location of the tumor without considering diffuse bile duct cancer, DIFFUSE in our study [5, 13]. However, our hypothesis was that the involvement of the middle BD itself is an important poor prognostic factor and tumors confined to the middle BD would have similar prognoses to diffuse BD cancer. Therefore, our classification and analysis (DISTAL vs MID/DIFFUSE) may be more desirable to show the poor prognosis of BD cancer with involvement in the extrapancreatic BD [14]. Additionally, we included only R0 resection in this study. Recently, Kamposioras K, et al. reported similar results with ours that common BD cancer involving extrapancreatic BD showed worse prognosis than tumor confined intrapancreatic BD due to higher frequency of R1 resection and venous resection [14]. Most patients of this study had worse tumor characteristics than present study, 74.6 % of patients had lymph node metastasis and R1 resection was done in 71.2 %. These advanced tumor status maybe weaken impact of tumor location that extent of tumor involvement was not independent prognostic factor. Generally, R1 resection has been known as an independent poor prognostic predictive factor [5, 9, 11, 26, 27]. Therefore, excluding R1 patients in the present study is valuable to show the real impact of extrapancreatic BD involvement on prognosis. Not only excluding R0, but also less lymph node involvement in present study may magnify impact of tumor location. Although we did not focus on pathological findings, present study clinically supports the result of Kamposioras K’s study.

In the tumor is confined to the middle BD, there have been debates about whether segmental BD resection is an adequate operative procedure or not [6]. A few studies have shown that similar incidence of positive surgical margin and similar long-term results between segmental BD resection and PD in the cancer confined to middle BD [7, 15, 26, 28]. Our results showed that the clinical characteristics and long-term results between MID and DIFFUSE were not different, and the long-term results according to the operative procedure, either BD resection or PD in these groups, were also similar (Fig. 4). With BD resection, sufficient radical lymph node dissection of group 2 (numbers 8, 12, and 13) is possible and enough radial margin can be obtained [28]. Although longitundial distal margin can obtain by PPPD, extent of radial margin is similar between PPPD and bile duct resection. Poor long-term results of tumors involving the middle BD may not due to the chosen operative procedure, but involvement of the middle BD itself. Therefore, in tumors confined to the mid BD without adjacent organ invasion, PD may have no survival benefit and segmental BD resection can be safely performed if R0 resection can be accomplished.

Our study showed poor prognosis of bile duct cancer involving extrapancreatic duct. However, this study was retrospective study with small patients number, therefore, further studies with larger cases are needed to validate it.

## Conclusion

In conclusion, extrahepatic cholangiocarcinoma involving the middle BD have a worse prognosis than those confined to intrapancreatic BD. In patients with tumors confined to the middle BD, BD resection can be an alternative surgical procedure to PD, if R0 resection can be accomplished.

## Abbreviations

BD: bile duct
PD: pancreaticoduodenectomy
